# Malignancy in ankylosing spondylitis: a cross-sectional analysis of a large population database

**DOI:** 10.1186/s41927-022-00275-x

**Published:** 2022-06-30

**Authors:** Mohamad Bittar, Sali Merjanah, Reem Alkilany, Marina Magrey

**Affiliations:** 1 Division of Connective Tissue Disease (Rheumatology), The University of Tennessee Health Science Center, Memphis, TN USA; 2 Division of Rheumatology, Boston University, Boston, MA USA; 3 Department of Medicine, The MetroHealth System Campus of Case Western Reserve University, Cleveland, OH USA; 4 Division of Rheumatology, University Hospitals/Case Western Reserve University, 11100 Euclid Ave, Cleveland, OH 44106 USA

**Keywords:** Ankylosing spondylitis, Axial spondyloarthritis, Malignancy, Cancer, Epidemiology

## Abstract

**Background:**

Increased cancer-risk has been reported with rheumatoid arthritis and systemic lupus erythematosus, but the risk is poorly studied in ankylosing spondylitis (AS). Conflicting data in AS have been reported in Asia and Europe, with lack of US population-based studies. Our objective is to study the prevalence of cancer in patients with AS in the US.

**Methods:**

Using the Explorys database, we performed a cross-sectional study. Data from AS patients and controls were stratified by 2 rheumatology visits, age groups, clinical characteristics, and frequency of cancers. The data were analyzed using a series of chi-square tests of independence as well as logistic regression to test for association between AS and cancer.

**Results:**

1410 AS patients (12.88%) had cancer. Female AS patients had a lower prevalence of cancer compared to controls (OR 0.840, 95% CI [0.769, 0.916]), while male AS patients had no statistically significant difference (OR 1.011, 95% CI [0.929, 1.099]). Among patients with AS, Skin cancers (squamous cell, malignant melanoma, and basal cell) and head and neck cancers were significantly increased.

**Conclusion:**

Our study demonstrated that the prevalence of “any-type-cancer” was not increased in AS patients compared to controls with no rheumatic disease. Skin, head, and neck cancers were more frequently seen in AS patients.

**Supplementary Information:**

The online version contains supplementary material available at 10.1186/s41927-022-00275-x.

## Key points

- Conflicting data about Malignancy risk in Ankylosing Spondylitis have been reported in Asia and Europe, with lack of US population-based studies.
- Our study demonstrated that the prevalence of “any-type-cancer” was not increased in AS patients compared to controls with no rheumatic disease.
- Skin, head, and neck cancers were more frequently seen in AS patients.

## Introduction

Ankylosing spondylitis (AS) is an inflammatory condition that predominantly affects the axial skeleton and is associated with several extra-articular manifestations like inflammatory eye, bowel, and skin diseases. The prevalence of AS varies widely between different continents and populations of the world, with the lowest prevalence being in Africa (~ 0.007% in some African countries) [1]. It is estimated that AS has a prevalence of 0.55% in the United States [2].

AS has been associated with co-morbidities like osteoporosis, fibromyalgia, cardiovascular disease, and cancer [3]. Few studies on malignancy risk in ankylosing spondylitis (AS) patients have provided conflicting data, with lack of US population-based studies. One systematic review and meta-analysis has shown a 14% increased risk of malignancy in AS (pooled RR 1.14; 95% CI 1.03–1.25) [4]. In another study of 6621 patients with AS, the overall cancer risk was not increased compared to the general population (SIR 1.05, 95% CI 0.94–1.17) [5]. Besides the role of inflammation, HLA B27 has been associated with increased risk of malignancies such as lymphoma and colorectal cancer [6, 7].

At present, there are no large-scale studies regarding the prevalence of cancer in patients with AS in the USA. A recent publication by Ward et al. [8] studied the risk of solid cancers in elderly patients with osteoarthritis or AS. The study captured AS Medicare beneficiaries (older than 65 years) but did not include younger age groups nor hematological malignancies. The objectives of this study were to look at the prevalence of solid and hematologic cancers in AS patients in the US.

## Methods

### Study design

This is a cross-sectional observational study using the IBM Explorys database [9], a pooled de-identified clinical database of > 60 million unique patients in the US. Explorys covers all 50 states, providing a broad regional distribution, and includes patients from different racial and socio-economic backgrounds. Explorys collects aggregated, standardized, and normalized clinical data from different electronic health records automatically updated in near real-time. In Explorys, patient records are mapped into a single set of Unified Medical Language System ontologies to facilitate searching and indexing. Diagnoses, findings, and procedures are mapped into the systematized nomenclature of medicine—clinical terms (SNOMED-CT) hierarchy.

Explorys matches the same patient across different healthcare systems and combines all their data by using a master patient identifier. All patient age cohorts are rounded to the nearest 10 years for de-identification purposes.

The study period was from January 2010 to January 2021. We collected data on AS patients and control subjects with no rheumatic diseases.

### Inclusion/exclusion criteria

We included 40–89-year-old AS patients with at least two visits with a rheumatologist and an ICD-10 diagnosis code of AS that is made during the rheumatology visits. The control group included adults 40–89 years of age with the exclusion of diagnosis of rheumatoid arthritis, systemic lupus erythematosus, psoriatic arthritis, systemic sclerosis, Sjogren’s, myositis or vasculitis, and at least one outpatient office visit during the study period. Patients were stratified based on 10-year age groups available in Explorys (group 1 40–49 years, group 2 50–59 years, group 3 60–69 years, group 4 70–79 years, group 5 80–89 years). For both groups, we excluded a previous diagnosis of cancer prior to January 2010. Date of cancer diagnosis was defined as the date of the first-ever malignant neoplastic disease diagnosis in the medical record after the qualifying AS diagnosis. All AS patients who met the inclusion criteria and had available information on the presence or absence of malignancy were included. After applying the exclusion criteria, the control group was randomly selected.

Data on the presence of hematologic and solid malignancies (colon, rectum and anus, head and neck, thyroid, lung, uterus, breast, melanoma, squamous cell carcinoma, basal cell carcinoma, prostate, bladder, leukemia, lymphomas) were recorded. We also collected data on sex (males/females), race (whites/blacks), and smoking status (active/ ex- and never smokers). Additional information on biologic use (tumor necrosis factor inhibitors and interleukin-17 inhibitors), the presence of inflammatory bowel disease, uveitis, and psoriasis were collected.

### Statistical analysis

Descriptive analysis included generating frequencies of the categorical variables. To address the primary research question, the data were first analyzed using a series of chi-square tests of independence to test for differences across the two groups (AS patients and controls) in the frequencies of cancers. Logistic regression analysis was then applied to adjust for important potential confounders such as age groups, sex, race, and smoking status. Based on the stratified data available, a maximum of two co-variates could be tested in one logistic regression model. Therefore, more than one logistic regression model was studied to adjust for different confounders. Variables that did not show to be confounding based on the regression analysis were excluded from the final model (for example variable race). Variables that had more than 25% missing data were studied in separate models but excluded from the final model (example variable smoking status). Odd-ratios of effect modifiers were reported separately. We also performed sensitivity analysis by excluding patients who received biologic treatments from the analysis. The statistical analysis was performed using SAS 9.4 software. Odds ratios, 95% confidence intervals, and *p* values were calculated and are reported. Threshold *p* values used were 0.05, below which the results were thought to be statistically significant.

This project received ethics board approval (IRB19-00404).

## Results

### Baseline characteristics

In the Explorys database, 12,810 AS patients were detected; information on the presence or absence of cancer was available for 10,940 patients. In this analysis, there were 10,940 patients with a diagnosis of AS and 13,344,160 controls. Of the AS patients included in the analysis, 5760 (52.7%) were females, 8900 (81.4%) were whites, and 2790 (25.5%) were active smokers. In the control group, 7,214,190 (54.1%) were females, 9,617,790 (72.1%) were whites, and 2,358,780 (17.7%) were active smokers. Psoriasis and uveitis were prevalent in 1780 (16.27%) and 1390 (12.7%) AS patients, respectively. Inflammatory bowel disease (IBD) was present in 560 AS patients (5.19%). Tumor necrosis factor inhibitors were used in 39.3% and IL-17 inhibitors in 4.48% of AS patients. In the control group, 185,260 (1.4%) patients had psoriasis, 45,270 (0.34%) patients had uveitis, and 115,990 (0.9%) patients had inflammatory bowel disease.

In the AS group, 1410 patients (12.88%) had a cancer diagnosis; 1210 (85.81%) of whom were white, 760 (53.9%) were males, and 430 (30.5%) were active smokers. In the control group, 2,055,530 patients (15.4%) had a diagnosis of cancer. Of these patients, 81.65% were white, 50.80% were females, and 21.25% were active smokers (Table 1). Data stratified by different age groups are available in the Additional file 1.

**Table 1:** Baseline characteristics of AS patients and controls (combined group 40–89 years)

Study group	Total AS N =	Missing N (%)	AS patients w/cancer N (%)	AS patients w/o cancer N (%)	Total control N =	Missing N (%)	Control w/cancer N (%)	Control w/o cancer N (%)
# of patients	10,940		1410 (12.9)	9530 (87.1)	13,344,160		2,055,530 (15.4)	11,288,630 (84.6)
Male	5180		760 (53.9)	4420 (46.4)	6,140,340		1,012,290 (49.2)	5,128,050 (45.4)
Female	5760		650 (46.1)	5110 (53.6)	7,214,190		1,044,170 (50.8)	6,170,020 (54.6)
White	8900		1210 (85.8)	7690 (80.7)	9,617,790		1,678,310 (81.7)	7,939,480 (70.3)
Black	940		90 (6.4)	850 (8.9)	1,321,400		169,780 (8.3)	1,151,620 (10.2)
Other races		1100 [10]				2,404,970 (18)		
Active Smoker	2790	3280 (29.9)	430 (30.5)	2360 (24.8)	2,358,780	7,255,250 (54.4)	436,780 (21.3)	1,922,000 (17.0)
Ex smoker	3630		650 (46.1)	2980 (31.3)	2,855,190		703,690 (34.2)	2,151,500 (19.1)
Never smoker	1240		250 (17.7)	990 (10.4)	874,940		209,000 (10.2)	665,940 (5.9)
TNFi	4300		530 (37.6)	3770 (39.6)	39,100		6110 (0.3)	32,990 (0.3)
IL-17i	490		60 (4.3)	430 (4.5)	2950		440 (0.02)	2510 (0.02)
IBD	560		120 (8.5)	440 (4.6)	115,990		27,990 (1.4)	88,000 (0.8)
Uveitis	1390		210 (14.9)	1180 (12.4)	45,270		9970 (0.5)	3530 (0.03)
Psoriasis	1780		200 (14.2)	1580 (16.6)	185,260		40,110 (1.95)	145,150 (1.3)

IBD: Inflammatory Bowel disease

TNFIs: Infliximab, Etanercept, Adalimumab, Golimumab, Certolizumab

IL-17i: Secukinumab, Ixekizumab

### Prevalence of malignancies: AS patients versus controls

Overall, AS patients had a lower prevalence of “any-type cancer” compared to controls (Table 2). This was consistent after adjusting for different potentially confounding variables including age groups, sex, race, and smoking status. After adjusting for age groups and sex, AS patients had 7.8% less odds to have cancer compared to controls (OR 0.922, 95% CI [0.868, 0.979]). After adjusting for age groups, female AS patients had 16% less odds to have cancer compared to controls (OR 0.840, 95% CI [0.769, 0.916]), while male AS patients had no statistically significant difference (OR 1.011, 95% CI [0.929, 1.099]).

**Table 2:** Odd-ratios of the association between AS and cancer based on different logistic regression models

	Odds ratio	95% CI	*p* value
Crude	0.813	[0.768, 0.859]	< 0.0001
Adjusted by age groups	0.931	[0.877, 0.989]	0.02
Adjusted by sex	0.811	[0.767, 0.858]	< 0.0001
Males	0.871	[0.806, 0.941]	
Females	0.752	[0.693, 0.816]	
Adjusted by race	0.743	[0.701, 0.787]	< 0.0001
Adjusted by smoking status	0.732	[0.690, 0.776]	< 0.0001
Adjusted by age groups and sex	**0.922**	**[0.868, 0.979]**	**0.0082**
Males	**1.011**	**[0.929, 1.099]**	
Females	**0.840**	**[0.769, 0.916]**	
Adjusted by age groups and race	0.845	[0.794, 0.900]	< 0.0001
Adjusted by age groups and smoking	0.881	[0.827, 0.939]	< 0.0001

Final model: adjusted by age groups and sex

The results in bold are the results of the final model we used, stratified by sex. Bold is significant as we made our conclusions based on these numbers

After subtracting patients who received biologic treatments from the total number of AS patients and controls, sensitivity analysis was performed, and no major difference was found. The new crude OR is 0.845 (95% CI [0.785, 0.909]), which is only ~ 3.8% different from the previous crude OR. After excluding patients who received biologic treatments, the new age-adjusted OR is 0.796 (95% CI [0.733, 0.864]), leading to similar conclusions. No sex-stratified data on biologic treatments available.

Among different types of cancers, AS patients had a higher prevalence of head and neck cancers compared to the control group (4.25% vs 2.54%, OR 1.70, 95% CI [1.32, 2.21], *p* value < 0.0001). Skin cancers were also more prevalent in the AS population studied. 7.09% of AS patients with cancer had malignant melanoma compared to 4.57% of the controls with cancer (OR 1.59, 95% CI [1.30, 1.95], *p* value < 0.0001); 9.22% of AS patients with cancer had squamous cell carcinoma of skin compared to 6.41% of the controls with cancer (OR 1.48, 95% CI [1.24, 1.78], *p* value < 0.0001); and 16.3% of AS patients with cancer had basal cell carcinoma compared to 12.58% of the controls with cancer (OR 1.35, 95% CI [1.18, 1.56], *p* value < 0.0001). The prevalence of colorectal, lung, breast, and prostate cancers were significantly lower in AS patients compared to the control group (Table 3). There was no statistically significant difference in the prevalence of leukemias, lymphomas, thyroid, uterine, and bladder cancers between the two groups. Other types of cancers like esophageal, stomach, liver, pancreas, ENT, brain, bone, cervical, testicular, and ovarian cancers were present in very low numbers in the AS group which did not achieve meaningful statistical significance. Stratifying individual cancers by age groups led to smaller numbers of cases in different groups which was not statistically meaningful.

**Table 3:** Comparing primary malignancies in AS patient and controls using chi-squared test

Primary cancer	Group (40–89)				
	AS with cancer N = (%)	Control with cancer N = (%)	Odds ratio	95% CI	*P* value
Colon	40 (2.8)	135,450 (6.6)	0.41	[0.30, 0.57]	< 0.0001
Rectum and anus	20 (1.4)	61,920 (3.0)	0.46	[0.30, 0.72]	0.0005
Head and neck	60 (4.3)	52,190 (2.5)	1.71	[1.32, 2.21]	< 0.0001
Thyroid	30 (2.1)	44,860 (2.2)	0.97	[0.68, 1.40]	0.888
Lung	60 (4.3)	127,710 (6.2)	0.67	[0.52, 0.87]	0.002
Breast	100 (7.1)	273,640 (13.3)	0.50	[0.41, 0.61]	< 0.0001
Malignant Melanoma	100 (7.1)	94,040 (4.6)	1.59	[1.30, 1.95]	< 0.0001
SCC	130 (9.2)	131,820 (6.4)	1.48	[1.24, 1.78]	< 0.0001
BCC	230 (16.3)	258,640 (12.6)	1.35	[1.18, 1.56]	< 0.0001
Bladder	30 (2.1)	59,590 (2.9)	0.73	[0.51, 1.05]	0.084
Leukemia	30 (2.1)	58,400 (2.8)	0.74	[0.52, 1.07]	0.107
Lymphoma	70 (5.0)	112,030 (5.5)	0.91	[0.71, 1.15]	0.422
Non-Hodgkins	60 (4.3)	90,810 (4.4)	0.96	[0.74, 1.25]	0.767

## Discussion

Using a large US healthcare database, the current study revealed that patients with AS do not have an increased prevalence of “any-type-cancer” compared to controls without a rheumatic disease. In the current study, among AS patients with cancer, the most prevalent cancers were mainly skin cancers, with basal cell carcinoma being the most common. Additionally, squamous cell carcinoma and malignant melanoma prevalence was also significantly higher in AS patients compared to non-AS patients. The only other group of malignancies that was present in a higher frequency in AS patients was head and neck cancer. This was in accordance with a Taiwanese study [10] that showed similar results. The risk of hematologic malignancies (leukemias, lymphomas) was not increased in AS patients in the current study compared to the general population. Similarly, the risk of Lung, GI and GU cancers was not increased in AS patients.

Unlike rheumatoid arthritis and systemic lupus erythematosus, at present, there are very few studies that have sought to investigate the risk of malignancy in AS. The results from the current study are similar to a previous Swedish population based cohort study [5] that included 6621 AS patients between 1965 and 1995; the authors concluded that the overall cancer risk was not increased in the cohort’s patients (SIR 1.05, 95% CI 0.94 to 1.17). An international study of prevalence of comorbidities in spondyloarthritis (ASAS-COMOSPA study [3] reported an overall prevalence for any type of cancer at 3.0% (95% CI 2.46 to 3.52), and the most prevalent cancer was cervical cancer (1.2% (95% CI 0.3 to 1.7)). The prevalence of basal cell carcinoma and melanoma were 0.8% (95% CI 0.6 to 1.2) and 0.7% (95% CI 0.4 to 1.0) respectively. Few studies from East Asia mainly Taiwan and Korea, have observed opposite results to the current study. A nationwide population-based retrospective cohort study from Taiwan [10] has revealed that, in 4133 AS patients, the overall risk of cancer was 38% higher than that in the general population with adjusted hazard ratio (HR) of 1.38, [95% confidence interval (CI) 1.18–1.60]. The risk was significantly higher with lung, head, and neck cancers. Another Taiwanese cohort [11] captured AS patients between 2000 and 2008 (sample size N = 5452); the results revealed that the overall incidence of cancer was elevated in AS [standardized incidence ratio (SIR), 1.15; 95% confidence interval (CI), 1.03–1.27]. There was an increased risk in hematologic malignancies and gender specific increases in prostate, bone, and colon cancer risk. A recent study from Korea [12] included only male patients (sample size n = 21,780) and concluded that the overall incidence of cancer was increased in AS patients (SIR 1.25, 95% CI 1.15–1.36), mainly reproductive system malignancies and pancreatic cancers. Additionally, a systematic review [4] was performed and showed a 14% (pooled RR 1.14; 95% CI 1.03–1.25) increase in the overall risk for malignancy, with patients from Asia being at the highest risk based on a sub-group analysis.

These conflicting data from the different studies are possibly impacted by race, genetics, and environmental factors. The aforementioned studies included homogenous patients of similar background, which makes the conclusions not widely generalizable.

At present, there are no large-scale studies regarding the prevalence of cancer in patients with AS in the USA. Ward et al. [8] studied the risk of solid cancers in elderly patients with osteoarthritis or AS, and it concluded that there was an increased risk of melanoma, GU and breast cancers; while the risks of GI and lung cancers were decreased.

A strength of our study is the use of a large clinical database comprising of a pooled de-identified information on > 60 million unique patients in the US. The Explorys database covers all 50 states, providing a broad regional distribution of source population from 26 major health systems representing both male and females of all age groups, different racial backgrounds throughout the US collected by thousands of clinicians as part of routine clinical care in inpatient and outpatient settings. Explorys uses a robust patient matching algorithm preventing information from being duplicated. The data are updated automatically at least once every 24 h [13]. This is the first study that provides information about the prevalence of any-type-cancer in patients with AS compared to controls in the USA. The study includes all ages, has racial diversity, and reports the risk of all malignancies, solid and hematologic.

One limitation of the study is that de-identified data at population level are available but further review of individual records is not possible. For example, we could stratify by 10-year age groups, but it was not possible to calculate the age mean. All patient age cohorts are rounded to the nearest 10 years for the purposes of de-identification. Using logistic regression, adjusting for more than two covariates in one model was not possible due to the nature of the collected data. It was possible to adjust for one or two covariates in one or multiple regression models. The Explorys search tool is limited to demographic information and other clinical information for which standard clinical informatics ontologies exist. In addition, there is variability of the length of time people are in the cohorts. The information about AS disease duration prior to the onset of cancer, duration of biologic use and family history of cancers were not available. Another limitation of our study is that some variables had > 25% missing data (ex. Smoking status), which can potentially confound the association with cancer. Data on some comorbidities (ex. cardiovascular disease) and exposures (ex. alcohol use) were not available, therefore these variables were not matched between AS patients and controls. Individual level data were not available to study the association of different variables (ex. TNF inhibitors) with cancers in patients with AS. This would be an important future study to determine what risk factors increase the risk of cancers in AS patients. To ensure provider utilization, we stratified our search to include at least two visits with a rheumatologist. To prevent duplication of patients, Explorys attempts to use a master-patient identifier and attempts to match the same patient across different healthcare systems and combines the data. However, there is still a possibility some patients may have been duplicated and encounters could have occurred outside the Explorys healthcare partners [14]. Ideally, a population based epidemiologic study is needed to look at the prevalence of cancer in AS patients, but would require funding, time and personnel. Explorys allows retrospective, large-scale studies with minimal resources. There are a very few studies in the literature that have evaluated the prevalence of cancer in patients with AS. To our knowledge, our study is the first real-world population study to look at prevalence of cancer in AS in the United States. Also, software platforms such as Explorys can provide a valid and useful method to investigate meaningful associations across large populations.

## Conclusion

Unlike other rheumatologic conditions, the prevalence of malignancy was not increased in patients with AS in the USA compared to controls with no rheumatic diseases based on this large population clinical database. It still needs to be determined, with certainty, what factors put patients at risk of developing cancer in the AS population, thus the need for large population-based studies to address this important question.

## Supplementary Information

**Additional file 1.** **Supplementary Table 1.** Baseline characteristics of AS patients and controls (group 1 40-49 years). **Supplementary Table 2.** Baseline characteristics of AS patients and controls (group 2 50-59 years). **Supplementary Table 3.** Baseline characteristics of AS patients and controls (group 3 60-69 years). **Supplementary Table 4.** Baseline characteristics of AS patients and controls (group 4 70-79 years). **Supplementary Table 5.** Baseline characteristics of AS patients and controls (group 5 80-89 years). **Supplementary Table 6.** Frequencies of different types of cancer in AS patients and controls, according to different age groups.

## Data Availability

The datasets generated or analyzed during this study are included in this published article.
