# Supplementary material for: Malignancy in ankylosing spondylitis: a cross-sectional analysis of a large population database
Source: BMC Rheumatol. 2022 Jun 30;6:44. doi: 10.1186/s41927-022-00275-x (PMC9245256; doi:10.1186/s41927-022-00275-x)
Supplement: Supplementary file 1 — Additional file 1. Supplementary Table 1. Baseline characteristics of AS patients and controls (group 1 40-49 years). Supplementary Table 2. Baseline characteristics of AS patients and controls (group 2 50-59 years). Supplementary Table 3. Baseline characteristics of AS patients and controls (group 3 60-69 years). Supplementary Table 4. Baseline characteristics of AS patients and controls (group 4 70-79 years). Supplementary Table 5. Baseline characteristics of AS patients and controls (group 5 80-89 years). Supplementary Table 6. Frequencies of different types of cancer in AS patients and controls, according to different age groups. [file 41927_2022_275_MOESM1_ESM.docx]

| Study Group | Total AS  N= | Missing  N (%) | AS patients w/ cancer N(%) | AS patients w/o cancer  N(%) | Total control N= | Missing N(%) | Control w/ cancer N (%) | Control w/o cancer N(%) |
| --- | --- | --- | --- | --- | --- | --- | --- | --- |
| # of patients | 1760 |  | 90 (5.1) | 1670 (94.9) | 1697970 |  | 92860  (5.5) | 1605110  (94.5) |
| Male | 820 |  | 40 (44.4) | 780 (46.7) | 740550 |  | 31680  (34) | 708870  (44) |
| Female | 950 |  | 50 (55.6) | 900 (53.9) | 961520 |  | 61480  (66) | 900040  (56) |
| White | 1350 |  | 70 (77.8) | 1280 (76.6) | 1141180 |  | 70990  (76.4) | 1070190  (66.7) |
| Black | 160 |  | 10 (11.1) | 150 (9.0) | 211130 |  | 8690  (9.4) | 202440  (12.6) |
| Other races |  | 250  (14.2%) |  |  |  | 345660  (20.4%) |  |  |
| Active smoker | 540 | 470  (26.7%) | 30 (33.3) | 510 (30.5) | 372940 | 916430  (54.0%) | 23540  (25.3) | 349400  (21.8) |
| Ex smoker | 540 |  | 30 (33.3) | 510 (30.5) | 286590 |  | 23930  (25.8) | 262660  (16.4) |
| Never smoker | 210 |  | 20 (22.2) | 190 (11.4) | 122010 |  | 11820  (12.7) | 110190  (6.9) |
| TNFi | 960 |  | 60 (66.7) | 900 (53.9) | 7370 |  | 580 (0.6) | 6790  (0.4) |
| IL-17i | 160 |  | 10 (11.1) | 150 (9.0) | 610 |  | 40  (0.04) | 570  (0.04) |
| IBD | 90 |  | 10 (11.1) | 80 (4.8) | 14740 |  | 1430  (1.5) | 13310  (0.8) |
| Uveitis | 300 |  | 20 (22.2) | 280 (16.8) | 5510 |  | 400  (0.4) | 5110  (0.3) |
| Psoriasis | 290 |  | 20 (22.2) | 270 (16.2) | 23540 |  | 1670  (1.8) | 21870  (1.4) |

Supplementary Table 1. Baseline characteristics of AS patients and controls (group 1 40-49 years).

IBD: Inflammatory Bowel disease

TNFIs: Infliximab, Etanercept, Adalimumab, Golimumab, Certolizumab

IL-17i: Secukinumab, Ixekizumab

In this group, 1890 AS patients were detected. 1760 AS patients who had available cancer information (presence or absence) were included in this analysis. Cells with “<10” observations generated by Explorys were adjusted to 10 in order to be included in the analysis.

| Study Group | Total AS  N= | Missing  N (%) | AS patients w/ cancer N(%) | AS patients w/o cancer  N(%) | Total control N= | Missing N(%) | Control w/ cancer N (%) | Control w/o cancer N(%) |
| --- | --- | --- | --- | --- | --- | --- | --- | --- |
| # of patients | 3200 |  | 230  (7.2) | 2970 (7.2) | 3418590 |  | 269520  (7.9) | 3149070  (92.1) |
| Male | 1490 |  | 100 (43.5) | 1390 (46.8) | 1562970 |  | 108840  (40) | 1454130  (46) |
| Female | 1710 |  | 130 (56.5) | 1580 (53.2) | 1860190 |  | 161050  (60) | 1699140  (54) |
| White | 2550 |  | 190 (82.6) | 2360 (79.5) | 2371640 |  | 212080  (78.7) | 2159560  (68.6) |
| Black | 290 |  | 20 (8.7) | 270 (9.1) | 382000 |  | 24670  (9.2) | 357330  (11.3) |
| Other races |  | 360 (11.3%) |  |  |  | 664950  (19.5%) |  |  |
| Active smoker | 910 | 1040  (32.5%) | 80 (34.8) | 830 (27.9) | 687780 | 1987540  (58.2%) | 67210  (24.9) | 620570  (19.7) |
| Ex smoker | 880 |  | 90 (39.1) | 790 (26.6) | 531330 |  | 67290  (25.0) | 464040  (14.7) |
| Never smoker | 370 |  | 40 (17.4) | 330 (11.1) | 211940 |  | 29650  (11.0) | 182290  (5.8) |
| TNFi | 1470 |  | 120 (52.2) | 1350 (45.5) | 12150 |  | 1320  (0.5) | 10830  (0.3) |
| IL-17i | 190 |  | 20 (8.7) | 170 (5.7) | 970 |  | 90  (0.03) | 880  (0.03) |
| IBD | 170 |  | 20 (8.7) | 150 (5.1) | 27380 |  | 3560  (1.3) | 23820  (0.8) |
| Uveitis | 450 |  | 50 (21.7) | 400 (13.5) | 9880 |  | 1200  (0.4) | 8680  (0.3) |
| Psoriasis | 520 |  | 40 (17.4) | 480 (16.2) | 44720 |  | 5080  (1.9) | 39640  (1.3) |

Supplementary Table 2. Baseline characteristics of AS patients and controls (group 2 50-59 years).

IBD: Inflammatory Bowel disease

TNFIs: Infliximab, Etanercept, Adalimumab, Golimumab, Certolizumab

IL-17i: Secukinumab, Ixekizumab

In this group, 3480 AS patients were detected. 3200 AS patients who had available cancer information (presence or absence) were included in this analysis. Cells with “<10” observations generated by Explorys were adjusted to 10 in order to be included in the analysis.

| Study Group | Total AS  N= | Missing  N (%) | AS patients w/ cancer N(%) | AS patients w/o cancer  N(%) | Total control N= | Missing N(%) | Control w/ cancer N (%) | Control w/o cancer N(%) |
| --- | --- | --- | --- | --- | --- | --- | --- | --- |
| # of patients | 3070 |  | 410  (13.4) | 2660  (86.8) | 3522930 |  | 470100  (13.3) | 3052830  (86.7) |
| Male | 1460 |  | 210  (51.2) | 1250  (47.0) | 1654550 |  | 225350  (48) | 1429200  (47) |
| Female | 1610 |  | 200  (48.8) | 1410  (53.0) | 1874180 |  | 245310  (52) | 1628870  (53) |
| White | 2530 |  | 350  (85.4) | 2180  (82.0) | 2555120 |  | 378920  (80.6) | 2176200  (71.3) |
| Black | 270 |  | 20 (4.9) | 250 (9.4) | 352370 |  | 42350  (9.0) | 310020  (10.2) |
| Other races |  | 270 (8.8%) |  |  |  | 615440  (17.5%) |  |  |
| Active smoker | 770 | 950  (30.9%) | 140 (34.1) | 630 (23.7) | 664950 | 1911920  (54.3%) | 113790  (24.2) | 551160  (18.1) |
| Ex smoker | 1020 |  | 180 (43.9) | 840 (31.6) | 728230 |  | 143180  (30.5) | 585050  (19.2) |
| Never smoker | 330 |  | 80 (19.5) | 250 (9.4) | 217830 |  | 46460  (9.9) | 171370  (5.6) |
| TNFi | 1190 |  | 180 (43.9) | 1010 (38.0) | 11170 |  | 1750  (0.4) | 9420  (0.3) |
| IL-17i | 120 |  | 20 (4.9) | 100 (3.8) | 940 |  | 160  (0.03) | 780  (0.03) |
| IBD | 160 |  | 40 (9.8) | 120 (4.5) | 28490 |  | 5880  (1.3) | 22610  (0.7) |
| Uveitis | 370 |  | 60 (14.6) | 310 (11.6) | 11570 |  | 2110  (0.4) | 9460  (0.3) |
| Psoriasis | 530 |  | 70 (17.1) | 460 (17.3) | 52580 |  | 10000  (2.1) | 42580  (1.4) |

Supplementary Table 3. Baseline characteristics of AS patients and controls (group 3 60-69 years).

IBD: Inflammatory Bowel disease

TNFIs: Infliximab, Etanercept, Adalimumab, Golimumab, Certolizumab

IL-17i: Secukinumab, Ixekizumab

In this group, 3540 AS patients were detected. 3070 AS patients who had available cancer information (presence or absence) were included in this analysis. Cells with “<10” observations generated by Explorys were adjusted to 10 in order to be included in the analysis.

| Study Group | Total AS  N= | Missing  N (%) | AS patients w/ cancer N(%) | AS patients w/o cancer  N(%) | Total control N= | Missing N(%) | Control w/ cancer N (%) | Control w/o cancer N(%) |
| --- | --- | --- | --- | --- | --- | --- | --- | --- |
| # of patients | 1930 |  | 350  (18.1) | 1580  (81.9) | 2562250 |  | 527200  (20.6) | 2035050  (79.4) |
| Male | 940 |  | 210  (60.0) | 730  (46.2) | 1202850 |  | 274860  (52) | 927990  (45.6) |
| Female | 990 |  | 130  (37.1) | 860  (54.4) | 1364980 |  | 252910  (48) | 1112070  (54.6) |
| White | 1650 |  | 300  (85.7) | 1350  (85.4) | 1910150 |  | 434560  (82.4) | 1475590  (72.5) |
| Black | 120 |  | 20 (5.7) | 100 (6.3) | 204710 |  | 39560  (7.5) | 165150  (8.1) |
| Other races |  | 160 (8.3%) |  |  |  | 447390  (17.5%) |  |  |
| Active smoker | 380 | 540 (28.0%) | 100 (28.6) | 280 (17.7) | 365900 | 1364080  (53.2%) | 103340  (19.6) | 262560  (12.9) |
| Ex smoker | 790 |  | 190 (54.3) | 600 (38.0) | 666610 |  | 190690  (36.2) | 475920  (23.4) |
| Never smoker | 220 |  | 60 (17.1) | 160 (10.1) | 165660 |  | 50720  (9.6) | 114940  (5.6) |
| TNFi | 530 |  | 110 (31.4) | 420 (26.6) | 6070 |  | 1410  (0.3) | 4660  (0.2) |
| IL-17i | 30 |  | 10 (2.9) | 20 (1.3) | 390 |  | 120  (0.02) | 270  (0.01) |
| IBD | 100 |  | 30 (8.6) | 70 (4.4) | 22750 |  | 6980  (1.3) | 15770  (0.8) |
| Uveitis | 220 |  | 60 (17.1) | 160 (10.1) | 9540 |  | 2600  (0.5) | 6940  (0.3) |
| Psoriasis | 340 |  | 40 (11.4) | 300 (19.0) | 39050 |  | 11500  (2.2) | 27550  (1.4) |

Supplementary Table 4. Baseline characteristics of AS patients and controls (group 4 70-79 years).

IBD: Inflammatory Bowel disease

TNFIs: Infliximab, Etanercept, Adalimumab, Golimumab, Certolizumab

IL-17i: Secukinumab, Ixekizumab

In this group, 2460 AS patients were detected. 1930 AS patients who had available cancer information (presence or absence) were included in this analysis. Cells with “<10” observations generated by Explorys were adjusted to 10 in order to be included in the analysis.

| Study Group | Total AS  N= | Missing  N (%) | AS patients w/ cancer N(%) | AS patients w/o cancer  N(%) | Total control N= | Missing N(%) | Control w/ cancer N (%) | Control w/o cancer N(%) |
| --- | --- | --- | --- | --- | --- | --- | --- | --- |
| # of patients | 620 |  | 160  (25.8) | 460  (74.2) | 1294710 |  | 339110  (26.2) | 955600  (73.8) |
| Male | 250 |  | 90 (56.3) | 160 (34.8) | 584410 |  | 179490  (53) | 404920  (42) |
| Female | 370 |  | 70 (43.8) | 300 (65.2) | 713210 |  | 159990  (47) | 553220  (58) |
| White | 540 |  | 150 (93.8) | 390 (84.8) | 976560 |  | 284500  (83.9) | 692060  (72.4) |
| Black | 50 |  | 10 (6.3) | 40 (8.7) | 88860 |  | 21240  (6.3) | 67620  (7.1) |
| Other races |  | 30 (4.8%) |  |  |  | 229290  (17.7%) |  |  |
| Active smoker | 100 | 180  (29.0%) | 30 (18.8) | 70 (15.2) | 137000 | 707320  (54.6%) | 51990  (15.3) | 85010  (8.9) |
| Ex smoker | 260 |  | 90 (56.3) | 170 (37.0) | 362650 |  | 129800  (38.3) | 232850  (24.4) |
| Never smoker | 80 |  | 30 (18.8) | 50 (10.9) | 87740 |  | 34520  (10.2) | 53220  (5.6) |
| TNFi | 120 |  | 40 (25.0) | 80 (17.4) | 1650 |  | 500 (0.1) | 1150  (0.1) |
| IL-17i | 0 |  | 0 (0) | 0 (0) | 60 |  | 30 (0.01) | 30 (0.003) |
| IBD | 40 |  | 20 (12.5) | 20 (4.3) | 12020 |  | 4630  (1.4) | 7390  (0.8) |
| Uveitis | 50 |  | 20 (12.5) | 30 (6.5) | 5010 |  | 1750  (0.5) | 3260  (0.3) |
| Psoriasis | 90 |  | 20 (12.5) | 70 (15.2) | 16330 |  | 6320  (1.9) | 10010  (1.0) |

Supplementary Table 5. Baseline characteristics of AS patients and controls (group 5 80-89 years).

IBD: Inflammatory Bowel disease

TNFIs: Infliximab, Etanercept, Adalimumab, Golimumab, Certolizumab

IL-17i: Secukinumab, Ixekizumab

In this group, 930 AS patients were detected. 620 AS patients who had available cancer information (presence or absence) were included in this analysis. Cells with “<10” observations generated by Explorys were adjusted to 10 in order to be included in the analysis.


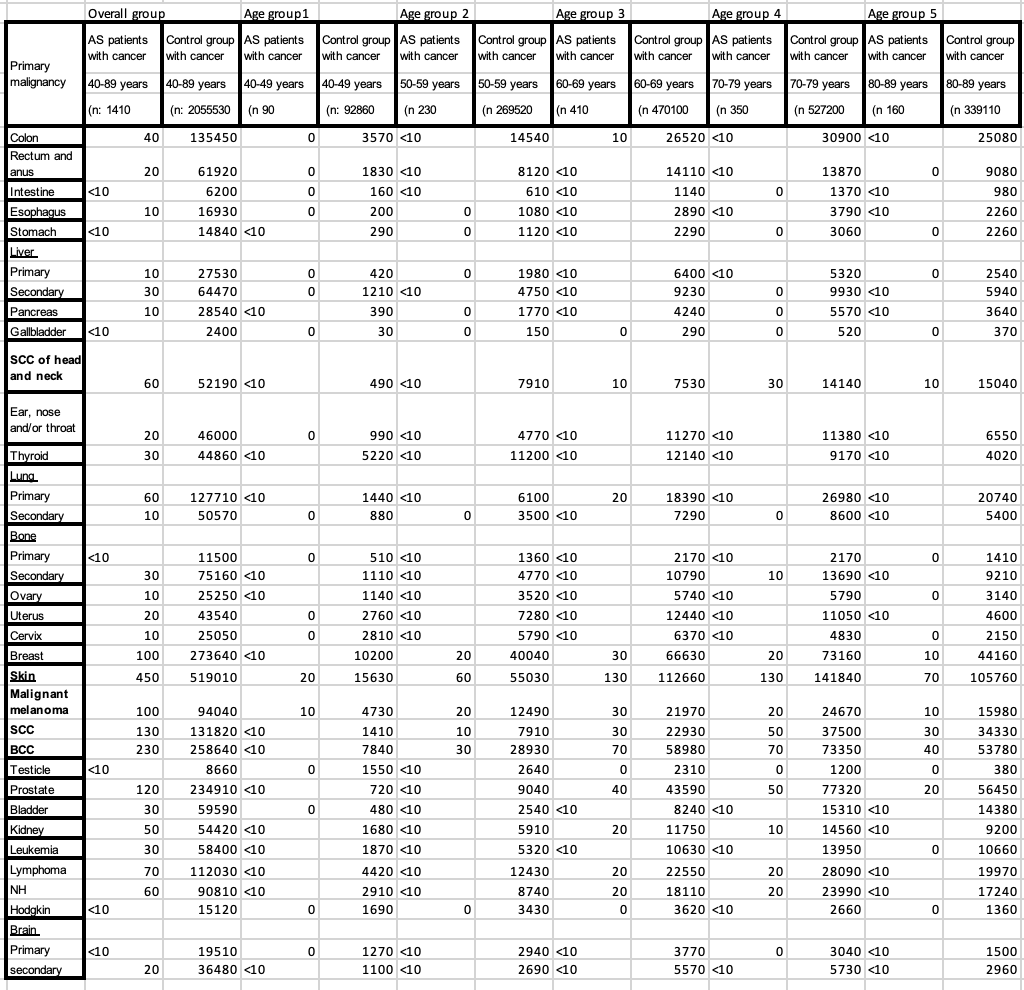


Supplementary Table 6. Frequencies of different types of cancer in AS patients and controls, according to different age groups.
